# Healthy Parent Carers: Acceptability and practicability of online delivery and learning through implementation by delivery partner organisations

**DOI:** 10.1111/hex.13812

**Published:** 2023-07-04

**Authors:** Alice Garrood, Gretchen Bjornstad, Aleksandra Borek, Annette Gillett, Jenny Lloyd, Sarah Brand, Mark Tarrant, Susan Ball, Annie Hawton, Annabel McDonald, Mary Fredlund, Fleur Boyle, Vashti Berry, Stuart Logan, Christopher Morris

**Affiliations:** ^1^ Peninsula Childhood Disability Research Unit (PenCRU) and NIHR Applied Research Collaboration South West Peninsula (PenARC) University of Exeter Medical School, University of Exeter Exeter UK; ^2^ NIHR Applied Research Collaboration South West Peninsula (PenARC) University of Exeter Exeter UK; ^3^ Nuffield Department of Primary Care Health Sciences, Medical Sciences Division University of Oxford, Radcliffe Observatory Quarter Oxford UK; ^4^ Relational Health Group and NIHR Applied Research Collaboration (PenARC) South West Peninsula, Department of Health and Community Sciences, Institute of Health Research, University of Exeter Medical School University of Exeter Exeter UK; ^5^ Health Economics Group and NIHR Applied Research Collaboration (PenARC) South West Peninsula University of Exeter Medical School, University of Exeter Exeter UK

**Keywords:** delivery partner organisations, disabled children, health promotion, implementation, parent carers

## Abstract

**Background:**

Parent carers of disabled children are at increased risk of physical and mental health problems. The Healthy Parent Carers (HPC) programme is a manualised peer‐led group‐based programme that aims to promote parent carer health and wellbeing. Previously, the programme had been delivered in person, with recruitment and delivery managed in a research context. This study explored implementation by two delivery partner organisations in the United Kingdom. Facilitator Training and Delivery Manuals were modified for online delivery using Zoom due to COVID‐19.

**Methods:**

The study methodology utilised the Replicating Effective Programs framework. A series of stakeholder workshops informed the development of the Implementation Logic Model and an Implementation Package. After delivering the programme, delivery partner organisations and facilitators participated in a workshop to discuss experiences of implementing the programme. A wider group of stakeholders, including commissioners, Parent Carer Forums and charity organisations representatives and researchers subsequently met to consider the sustainability and potential barriers to delivering the programme outside the research context.

**Results:**

This study explored implementation by two delivery partner organisations in the United Kingdom that were able to recruit facilitators, who we trained, and they recruited participants and delivered the programme to parent carers in different localities using Zoom. The co‐created Implementation Logic Model and Implementation Package were subsequently refined to enable the further roll‐out of the programme with other delivery partner organisations.

**Conclusions:**

This study provides insight and understanding of how the HPC programme can be implemented sustainably outside of the research context. Further research will evaluate the effectiveness of the programme and refine the implementation processes.

**Patient and Public Contribution:**

Parent carers, delivery partner organisation staff and service commissioners were consulted on the design, delivery and reporting of the research.

## 1 INTRODUCTION

Parent carers of disabled children are at increased risk of physical and mental health problems.^1^, ^2^, ^3^, ^4^, ^5^, ^6^, ^7^, ^8^, ^9^, ^10^, ^11^, ^12^, ^13^ They often experience greater challenges in maintaining good personal health, which has implications for their own wellbeing and their ability to care for their children.^14^ Individual, family and environmental factors affect parent carers' health. Social disadvantage, gender, ethnicity, sexual orientation and/or other personal factors may intersect to increase the health impacts of being a parent carer.^15^ Population‐based studies suggest that parent carer health problems persist and may worsen over time.^3^ The COVID‐19 pandemic exacerbated this problem, disproportionately affecting parent carers, with lockdowns, school closures and limited services leaving many families feeling abandoned.^16^, ^17^

Our consultations with parent carers suggest existing public health interventions are perceived as insensitive to the challenges that parent carers experience. Interventions to promote health equity are urgently needed.^18^ The Healthy Parent Carers (HPC) programme was developed specifically to promote the health and wellbeing of parent carers. It aims to improve health and wellbeing by engagement in behaviours associated with better health— Connect, Learn, be Active, take Notice, Give, Eat well, Relax, Sleep (CLANGERS). Intervention development and programme components and delivery strategies were described comprehensively in our previous papers.^19^, ^20^

**Figure 1:**
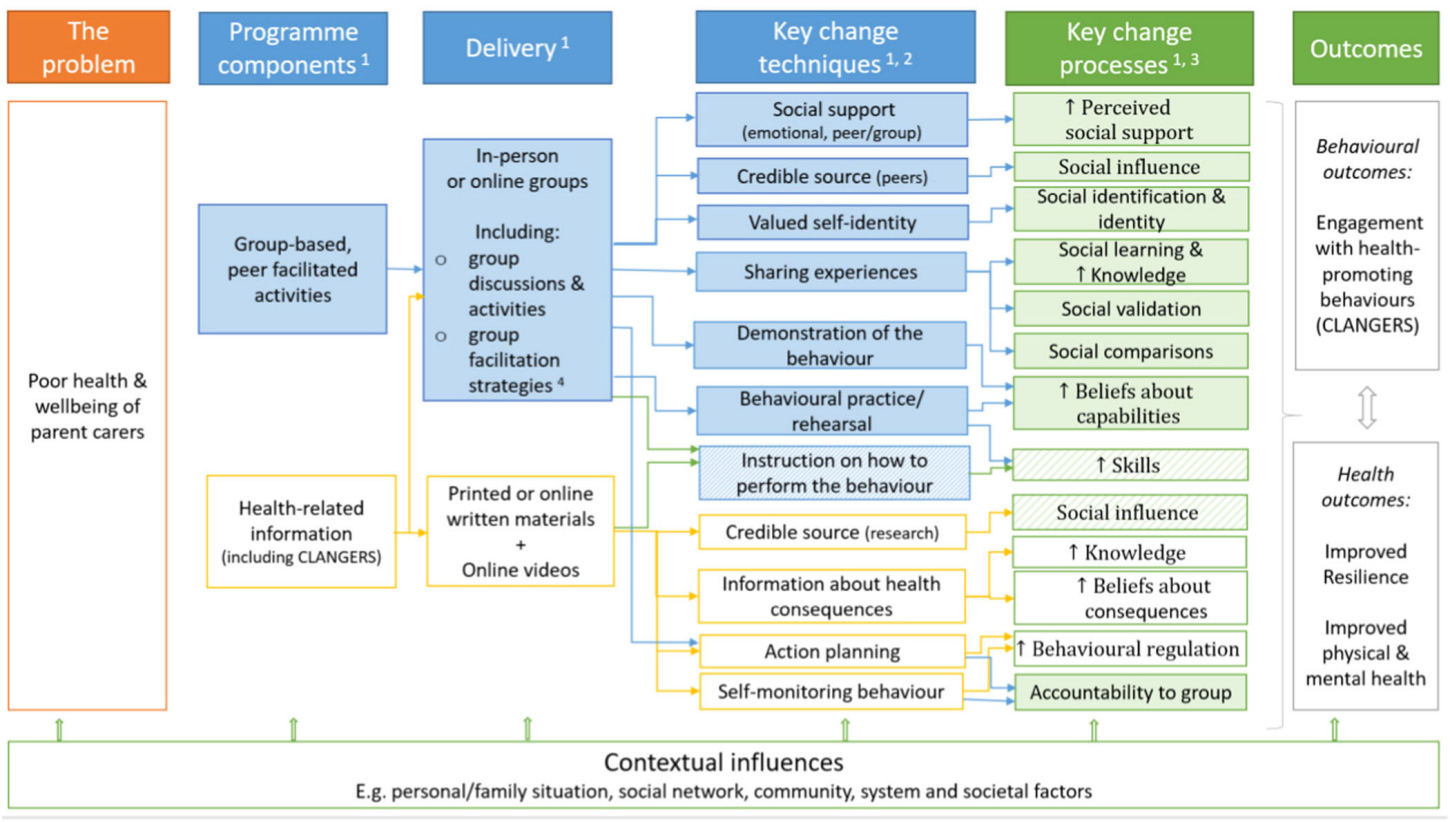
Intervention logic model for the Healthy Parent Carers programme (v.2). 1. Colour‐filled boxes indicate the components, techniques and processes that are specific to a group‐based delivery, whereas the white‐filled boxes indicate those present in the self‐directed delivery (i.e., when participants use the written and online resources). Pattern‐filled boxes indicate change processes that can be present in both group and self‐directed delivery but are likely to be reinforced in a group setting. 2. Include the key/core behaviour change techniques (BCTs) to facilitate intrapersonal change processes (based on the BCT taxonomy v1^21^ and to facilitate interpersonal change processes (based on the MAGI framework).^22^ 3. Include the key/core intra‐ and interpersonal change processes in behavioural determinants. The determinants are drawn from the Theoretical Domains Framework^23^ and interpersonal change processes are drawn from the MAGI framework.^22^ 4. Include strategies to facilitate a conducive group environment (e.g., ice‐breakers), group activities and discussions, and change techniques in groups. CLANGERS, Connect, Learn, be Active, take Notice, Give, Eat well, Relax, Sleep; MAGI, Mechanisms of Action in Group‐Based Interventions.

The updated intervention logic model of the HPC programme outlines that parent carer engagement with health‐promoting activities (CLANGERS) will foster resilience and improve health and wellbeing (Figure 1). The programme facilitates behaviour change by providing opportunities for and prompting, social (peer) support, development of a shared social identity, sharing of experiences and the practice of health‐related behaviours. This is achieved through (i) facilitated group‐based activities and discussions, and (ii) providing health‐related information and resources.

The HPC programme is delivered to groups of 6–12 parent carers, led by pairs of trained peer Lead and Assistant Facilitators, following procedures in the Facilitator Delivery Manual. Participants also receive written materials (printed/online), mirroring the content discussed in groups, to refer to and use outside of the group sessions. These include information about CLANGERS, links to videos and useful resources and action planning and self‐monitoring sheets.

Previously, researchers recruited facilitators, set up delivery sites, advertised for and screened potential participants, prepared resources and supported facilitators during delivery. However, outside of the research context, these tasks need to be done by licensed delivery partner organisations. The transition from academic to real‐world settings is a common challenge for many evidence‐based interventions.^24^, ^25^ The present study was, therefore, designed to establish and test a strategy to enable successful implementation by delivery partner organisations, which includes charities, social enterprises and voluntary groups.

Although the HPC programme has not yet undergone a definitive effectiveness trial, we wanted to explore barriers to implementation in nonacademic community settings, to ensure as early as possible that the intervention (if proven effective) would be implementable. The HPC programme is a complex intervention by virtue of the range of behaviours targeted, expertise and skills required by those delivering the intervention and other programme contextual factors. The recent Medical Research Council framework for complex interventions highlighted the nonlinearity of addressing important questions related to feasibility, effectiveness and implementation of interventions.^26^ Thus, following intervention development and a feasibility study,^19^, ^27^ we considered it critical to explore potentially feasible and sustainable implementation strategies. This is particularly important as reaching vulnerable individuals and those facing health inequalities presents numerous unique issues which are not well documented.^28^ This paper reports on the first steps towards translating the HPC programme to delivery in real‐world settings, preceding a pragmatic evaluation of effectiveness.

The study was designed before the Covid‐19 pandemic. Social distancing mitigations in the pandemic meant that HPC could not be delivered in person, as initially designed. It was necessary to first adapt the programme (before implementation) so that training of facilitators and delivery of the programme could be done remotely using Zoom™. Details of the adaptations to online delivery are reported in Supporting Information: File 1. Therefore, this study serendipitously enabled our first evaluation of the acceptability and practicability of online delivery.

This implementation study had the following aims:

1. To identify feasible and acceptable strategies for wider implementation of HPC with delivery partner organisations from the perspective of the organisations, facilitators and participants.
2. To explore barriers and enablers for implementation of the programme by two delivery partner organisations who work with families with disabled children.
3. To systematically develop and refine the implementation strategy, including the Implementation Logic Model, Implementation Package and the terms for future licensing, to optimise the programme for delivery with nonacademic organisations.

Additionally, due to the need to move the facilitator training and the HPC programme online, we explored the acceptability and practicability of online delivery.

## 2 METHODS

The Replicating Effective Programs (REP) framework was developed specifically to provide a systematic process for implementing health interventions outside academic settings by community‐based organisations.^29^ The framework aims to help maintain fidelity while maximising the transferability of interventions when they are translated from academic to community settings. As this was our aim of exploring the delivery of HPC by community‐based delivery partner organisations, the REP was considered a particularly relevant framework. The REP framework consists of four phases: preconditions, preimplementation, implementation and maintenance and evolution. This study focused on the first two stages outlined by the framework (Table 1).

**Table 1:** Study method mapped to REP phases 1 and 2.

Task	Activity	Output
*Phase 1—preconditions*		
Identify barriers	Establish a Community Working Group including representatives from Peninsula Childhood Disability Research Unit, Public and Patient Involvement groups; delivery partner organisations. Workshop 1—orientation meeting.	Partnership building between intervention developers and delivery organisations. Identified barriers and processes of implementation. Developed Implementation Logic Model.
Identify need	Funder workshop—determine local need and appetite for commissioning HPC.	Identified interest from commissioners and possible challenges to programme delivery.
Draft implementation	Draft implementation processes—in collaboration with delivery organisations and key stakeholders.	Co‐created a draft Implementation Package and Logic Model.
*Phase 2—preimplementation*		
Optimising implementation	Workshop 2—further refinement of the Implementation Package and Logic Model, including costs and any data that will need to be collected.	Implementation package reviewed. Contents developed to include additional key processes.
Pilot test	Train facilitators to deliver programme. Pilot test implementation and delivery. Parent carers take part in two pilot groups (6 or 12 weeks in length), implemented by delivery organisations.	Piloted the Implementation Package.
Evaluate and reflect	Workshop 3—review experiences of staff from delivery organisations and strategies undertaken during pilot testing. Workshop 4—discuss with potential future funders around sustainability and roll‐out of the programme.	Identified key roles, processes and knowledge required to implement the programme. Revised the Implementation Logic Model. Developed a greater understanding of possible challenges and appetite for delivering the programme in different organisations.

Abbreviations: HPC, Healthy Parent Carers; REP, Replicating Effective Program.

We collaborated with two national organisations as delivery partners. The Council for Disabled Children (CDC) is the umbrella body for over 300 voluntary and community organisations in England. Contact carries out a range of activities supporting families with disabled children in the United Kingdom. Both organisations are commissioned to deliver programmes for the Department of Education and/or the Department of Health and Social Care. Both organisations offer a range of training, support services and consultancy to parent carers, health professionals, social workers, local authorities and service providers in the childhood disability sector. Therefore, they were perceived as having the right reach, infrastructure and connections to implement the HPC programme.

We also continued to work in partnership with parent carers in our Family Faculty Patient and Public Involvement group who advise on our research. A series of meetings were coordinated to support the adaption of the programme for delivery online and reflect on the findings from implementation.

We established a Community Working Group (CWG). Delivery partner organisations selected key personnel to attend based on their knowledge of who would be able to support the implementation of the programme within their organisations. Two parent carer co‐investigators attended the meetings. Both had been involved in the programme and its development since the start and therefore could share their knowledge and expertise about the programme. One parent carer who had been a facilitator in the feasibility trial also took part. They were invited as they had also been previously employed by both of the delivery partner organisations as a facilitator. All co‐investigators were invited to attend the group meetings.

### 2.1 REP phase 1: Preconditions: Identifying barriers, need and drafting implementation package

In workshop 1, we introduced the HPC programme and our previous research to the new delivery partners. Discussion centred around necessary delivery tasks including site set‐up, recruitment of Lead and Assistant Facilitators, training of facilitators, preparation of training materials, recruiting participants, preparing delivery materials, orientation for facilitators, liaising with facilitators and participants, supervision, administration and facilitating delivery support calls. Delivery partners then presented how they delivered comparable programmes within their own organisations. The group also discussed sustainability, quality assurance, safeguarding and signposting.

### 2.2 REP phase 2: Preimplementation: Optimising implementation

The preimplementation stage involved pilot testing the package of the Implementation Manual, license agreement, contracts, Online Facilitator Training Manual, Online Delivery Manual, and management of postdelivery support calls.

Workshop 2 with the CWG involved discussions on the proposed Implementation Package, including implementation costs, which data would need to be collected, and the terms of a licensing agreement. Devising the Implementation Package involved key roles and responsibilities and the specific personnel who would be able to fulfil these roles within the Delivery partner organisations.

### 2.3 REP phase 2: Pilot testing the implementation

#### 2.3.1 Facilitator recruitment and consent

Delivery partner organisations identified parent carers to train as Lead and Assistant Facilitators using our predefined person specifications for each role.

Prospective peer facilitators were screened by a researcher and invited to document consent for participating in the study. They completed a pretraining baseline questionnaire, which included age, sex, motivations to be a facilitator, relevant experience and expectations of delivering the programme.

#### 2.3.2 Facilitator training

Trainers followed the Online Training Manual to train new facilitators. Trainee facilitators attended a 1‐h, pretraining session with the two trainers and study coordinator, which allowed everyone to meet as a group and to be briefed about the study and training. The session prepared facilitators for the online aspects of the programmes, which included specific online software: Zoom™ (video‐calling) functions and methods of using Jamboard™ (online whiteboard). The training was delivered in two ‘Blocks’. Each block consisted of 3 days of training in total. Block 1 was just for Lead Facilitators, and Block 2 was for Lead and Assistant Facilitators. Each Lead and Assistant Facilitator was given their own copy of the Online Delivery Manual to support their training and subsequent delivery.

#### 2.3.3 HPC programme participant recruitment and consent

We shared an exemplar advert which organisations used to advertise the programme. The advert was adapted by organisations to include specific information on the times, dates and contact details of their organisation. Their advertising strategies sought to reach a diverse range of parent carers. These included utilising local contacts and organisations, such as voluntary and community partners, local education authorities, health and statutory services, support groups for parent carers and social media, including Parent Carer Forums (www.nnpcf.org) on Facebook or Instagram.

Both delivery partners used the Eventbrite™ online platform for potential participants to register expressions of interest.

People who registered interest were initially contacted by a member of the organisation to complete a screening call and confirm eligibility and understanding. Participants' contact details were uploaded onto a password‐protected screening spreadsheet to track screening and recruitment.

Eligible parent carers were then invited to a screening meeting with a researcher to learn about the research aspects and what participation would entail and to check participants' familiarity with and access to Zoom™. Participants could opt out of research participation and still participate in the HPC group. If participants were happy to take part in the research, the researcher emailed a copy of the Participant Information Sheet ahead of a subsequent meeting to document consent.

#### 2.3.4 HPC programme delivery

Both delivery partner organisations delivered the 12 HPC programme modules through 2‐h online group sessions, twice per week, over a 6‐week period. The two courses were run in two separate localities in England, with participants and facilitators recruited from two different areas, one rural and one urban. Contact ran a daytime course, which took place at the same time and days each week. CDC ran a mix of day and evening sessions, which took place on the same days weekly. After each session, facilitators completed attendance registers and self‐reported fidelity checklists to indicate the specific content they covered (adherence), the duration of the sessions (dose) and parent/carer engagement (responsiveness). A minimum of 6–8, and a maximum of 12, participants per group were suggested based on findings from the feasibility trial.^27^

### 2.4 REP phase 2: Evaluate and reflect

#### 2.4.1 HPC programme participant baseline measures

After consent, participants were emailed a link to the baseline questionnaire, which asked questions on parent carer demographics and the About My Child (AMC) questionnaire.^30^ The AMC is a valid tool that measures the impact and complexity of the disabled child's medical condition. For this study, we used impact scores. Scoring ranged between 0 and 19, with higher scores indicating a greater impact. A £25 electronic shopping voucher was emailed to the programme participants on completion of the baseline and end‐of‐programme feedback forms.

#### 2.4.2 HPC programme participant feedback forms

Participants in the HPC programme who consented to take part in the research were emailed a link to the End of Programme Feedback Questionnaire as a secure *Microsoft Form* during their final session. The questions asked for information about how they heard about the programme, the course delivery and their experiences of participation. Questions were asked about attendance and any reasons or perceived barriers to this.

As this was the first time the programme had been delivered online, it was particularly important for us to seek participants' experiences of this delivery format. Therefore, questions were asked about online facilitation and any barriers to accessing online platforms.

#### 2.4.3 Stakeholder workshop 3

CWG members and facilitators both attended workshop 3, which aimed to gain postimplementation insight into the roles, expectations, gaps and tasks involved in the implementation of the HPC programme.

Workshop attendants were divided into two small groups that met online on different days. Groups comprised a mix of roles from both organisations. Roles included a senior manager, responsible for the strategic and budgetary decisions; personnel who coordinated the day‐to‐day delivery tasks, Lead and Assistant Facilitators, trainers and researchers. The interactive group‐based format of the workshop allowed for greater cross‐role discussions into the challenges of implementation led by nonprofit organisations. Experienced qualitative researchers facilitated the workshops.

The workshops explored the key roles, processes and knowledge required to implement the programme. During the workshop, we used the online platform MIRO™ which is a large, interactive and collaborative board to collect data. Those attending the workshop could simultaneously add notes, discussions and diagrams to the Miro board. Postworkshop, the data were organised into different categories based on what they referred to.

#### 2.4.4 Stakeholder workshop 4

Workshop 4 focused on longer‐term sustainability strategies. Members of the CWG met with commissioners, representatives from other delivery partner organisations and Parent Carer Forums to discuss potential wider rollout. These new stakeholders were engaged in a consultation capacity and their consent for research was not formally documented. The agenda included core themes for discussion based on information generated from workshop 3 and pilot testing, including sustainability, and hopes and barriers to delivering the programme in the future. The workshop was facilitated by the study's principal investigator. Members of the study team met afterwards to identify core learning and themes from the workshop.

#### 2.4.5 Analyses

Descriptive statistics were used to describe the flow of HPC participants through the study, summarise baseline demographics, baseline scores for the AMC,^30^ and responses to follow up. SPSS was used to analyse descriptive statistics, including the numbers and percentages of HPC participants choosing each response option in the follow‐up feedback questionnaire.

Qualitative data collected in stakeholder workshops, including notes on the Miro board and field/meeting notes, were analysed descriptively. We used pragmatic content analysis and sorted the comments and quotes into categories relating to different aspects of the implementation.

## 3 RESULTS

Across the series of four workshops, a range of personnel attended (Table 2).

**Table 2:** Role and number of personnel attending each workshop.

Role	Workshop 1	Workshop 2	Workshop 3	Workshop 4
Delivery partner manager/coordinator	2	3	4	3
Lead/Assistant Facilitators	2	2	6	1
Research team/co‐investigator	7	6	4	3
Business Development Manager University of Exeter	0	1	0	1
Parent Carer Forum representative	0	0	0	2
Commissioner/funder	0	0	0	4

### 3.1 REP phase 1: Preconditions: Identifying barriers, need and drafting implementation package

Workshop 1 was attended by members of the CWG. Members gained a shared understanding of similarities and differences in terms of the set‐up and training needs for the HPC compared to similar programmes run by partner organisations. This enabled the implementation package to be developed and possible gaps in training and resources identified. After this workshop, a draft of the Implementation Logic Model was developed. The model was further refined after each workshop.

### 3.2 REP phase 2: Optimising implementation

Members of the CWG met again in workshop 2 to discuss the proposed Implementation Package and add additional information on costings, data collection and the terms of a licensing agreement. Key roles and responsibilities were assigned to specific personnel, these included advertising, preparation of materials, support calls and supervision. Specification for the Lead role includes having knowledge and understanding of the issues affecting disabled children, young people and their families and the key challenges that parent carers face; the ability to work with parents in a sensitive and empathic way and experience in delivering training or support to others. Assistant Facilitator role descriptors include knowledge and understanding of how being a parent carer can impact on personal health and wellbeing, and experiences or aspirations to improve the health and wellbeing of other parent carers. Four freelance facilitators (two Lead Facilitators and two Assistant Facilitators) were recruited by the delivery partners and they consented to take part in the research. Facilitator characteristics were collected via the pretraining baseline questionnaire, which included age, sex, motivations to be a facilitator, relevant experience and expectations of delivering the programme.

### 3.3 REP phase 2: Pilot testing of the implementation

#### 3.3.1 Facilitator recruitment and training

All facilitators approached by the organisations agreed to participate. Contact trained a ‘reserve’ Lead Facilitator, who also participated in the CWG. These five facilitators completed the online training to deliver the programme. Lead Facilitators attended 36 h of online training, and Assistant Facilitators attended 18 h in total. The training was delivered in two ‘Blocks’. Each block consisted of 3 days of training. Block 1 was just for Lead Facilitators, and Block 2 was attended by all facilitators.

### 3.4 HPC programme participant demographics and feedback

#### 3.4.1 HPC programme participants' demographics

Twenty parent carers expressed an interest in the programme (Contact *n* = 10; CDC *n* = 10). Three parent carers did not respond to further contact after initial enquiries; 17 were formally assessed for eligibility (Contact = 9; CDC = 8). One programme participant (from CDC) was unable to attend the programme at the available dates or times. All 16 remaining participants consented to participate in the research and completed baseline questionnaires. Fifteen participants completed a follow‐up questionnaire after the programme finished. One programme participant withdrew after the group had started but remained in the study and was able to complete the follow‐up questionnaire.

**Figure 2:**
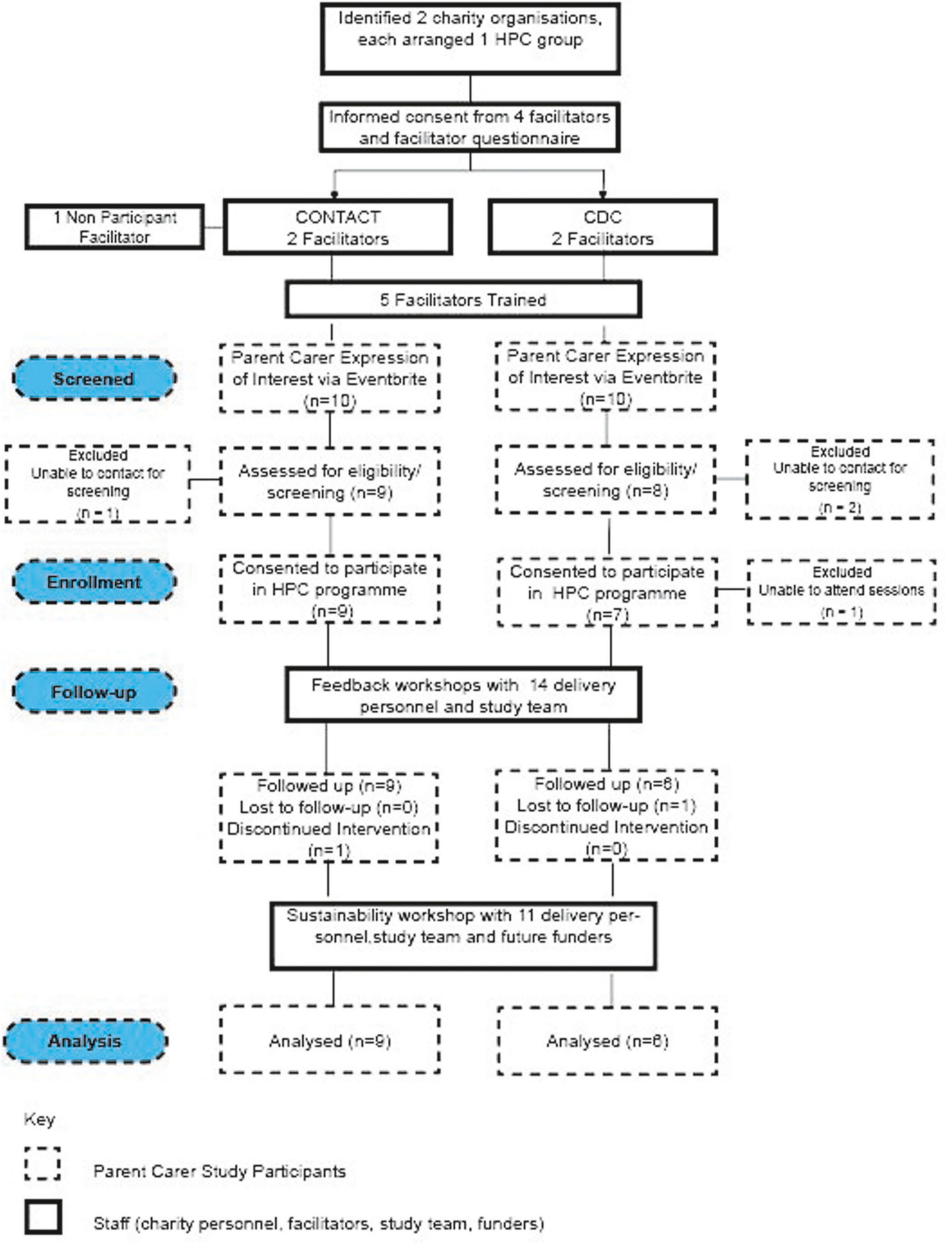
Study design and flow of HPC participants. CDC, Council for Disabled Children; HPC, Healthy Parent Carers.

Programme participants had a mean (SD) age of 44.1 (3.9) years, 15/16 (94%) were female, 6/16 (38%) were Asian/Asian British, 6/16 (38%) were White and 4/16 (25%) were Black/African/Caribbean/Black British. Sixty‐three percent (10/16) of participants were married, or in a civil partnership, 11/16 (69%) were unemployed and 10/16 (63%) had two or more Advanced‐Level qualifications (recognised for entrance to higher education) or above. Nineteen percent (3/16) of participants lived in a postcode ranked in the most deprived quintile based on the index of Multiple Deprivation 2019.^31^ The total mean score for participant's index child on the AMC was 53, with a range of 37. Participants' demographic characteristics at baseline are summarised in Supporting Information Materials: Table S1. Figure 2 illustrates the study design and the flow of participants.

#### 3.4.2 HPC programme participants' feedback

Fifteen participants who completed the end‐of‐programme questionnaire reported hearing about the study via Parent Carer Forums (*n* = 5), social media/word of mouth (*n* = 6) or via delivery organisations (*n* = 3). All reported that the initial pre‐meeting with the facilitator was helpful in making them feel comfortable to attend. Most participants, 14/15 (93%), were happy to access an online group, were satisfied with how the programme was delivered and reported that they found it useful in helping them to improve their health and wellbeing. Sixty percent (9/15) of participants stated that they would not have been able to attend an in‐person group. All respondents stated that they would recommend this programme and felt included and part of the group.

Ninety‐three percent (14/15) of participants did not experience any issues with their internet connection during the programme. Eighty‐seven percent (13/15) of participants felt that the date and times of the sessions were fine, and 11/15 (73%) felt that the length of the sessions was about right. Ninety‐three percent (14/15) of participants said that they were able to, and confident in accessing the online platforms used in the groups (Zoom™, JamBoard™).

Ninety‐three percent (14/15) stated that they had missed a group session, with 7/16 (44%) specifically reporting that they attended 10/12 sessions in total. All participants reported attending at least one session. The most commonly reported reasons for missing sessions were due to work commitments (*n* = 3), illness/medical appointments (*n* = 4) and caring responsibilities (*n* = 5).

### 3.5 Workshops 3: Delivery partners' experiences of implementing HPC

During workshop 3, key considerations for implementation were identified. These included coordination and administrative roles to support implementation activities and the acknowledgement of the time commitment required by staff from delivery organisations. Furthermore, online delivery was perceived to enhance accessibility by reaching more people more expediently and inexpensively. Specific topics raised in this workshop included: (i) key aspects of successful implementation, (ii) the specific resources the research team utilised to achieve this, (iii) what, if any, equivalents the delivery partners had to achieve this or (iv) if there were any potential barriers or different ways to deliver within different contexts.

Clear roles within the delivery organisations were also identified around the different tasks involved in implementation; these included: strategic management, to support the integration within the organisation, identify staff and costs to support the delivery of the programme; Project Management, to identify facilitators and coordinate programme set up and recruitment; Supervisors, who have knowledge of the programme but also are skilled in supporting with risk and the emotional wellbeing of the facilitators where required; Administration, to support with posting and printing resources, setting up Eventbrite and calendar invites for programme participants to attend groups; Trainers, who are skilled facilitators, with in‐depth knowledge of the programme; Lead Facilitators, who are experienced, with the required level of skills to support parent carers and Assistant Facilitators, with some knowledge and understanding of how being a parent carer can impact on personal health and wellbeing. Four categories related to roles and tasks were identified: Coordination of the Programme; Knowledge of the Programme; Governance and Strategic Direction (Box 1).

Box 1: **Specific tasks, knowledge and skills required for delivery, identified by participants in workshop 3**

Tasks/roles

‐ Coordinating admin tasks/streamline admin processes
‐ Choosing dates
‐ Design recruitment plan
‐ Design advert/marketing
‐ Arrange supervision
‐ Supporting the facilitators
‐ In‐depth understanding of the programme
‐ Set up and monitor Eventbrite™
‐ Contacting parent‐carers who sign up
‐ Send out Zoom™ links
‐ Emailing resources to facilitators
‐ Printing/getting quotes for printing
‐ Arranging support calls

Knowledge of programme

‐ Identifying appropriate facilitators—with necessary skills and competencies
‐ Knowing what the facilitator needs

Governance (safeguarding, GDPR, quality assurance)

‐ Quality assurance of delivery and training
‐ Ensure the knowledge of the programme is and stays ‘in house’
‐ Memorandum of Understanding—adding detail to the manualised template relevant to Delivery partner organisations (facilitator: organisation; funder: organisation)
‐ Managing consent/GDPR considerations/GDPR considerations and control
‐ Monitor/communicate safeguarding/safeguarding training—need shared understanding of this—piece of work to agree on this
‐ Monitoring and evaluating—reporting and collation
‐ Insurance and DBS up to date
‐ Considering intellectual property, not for profit sharing but maintaining quality
‐ Accountable for safeguarding policies
‐ Trademark/Certificate of facilitation/recognise delivery programme)

Strategic direction (funding, staffing negotiation)

‐ Determine if fundable/secure funding to support delivery/negotiate with funder/monitor if self‐sustainable
‐ Make decisions about how much money to spend
‐ Create staff capacity
‐ Negotiate payments/agree on rates for facilitators
‐ Prioritising and opportunity/cost planning: Situate within strategic aims (and is it fundable/deliverable)
‐ Strategic planning

As a result of this workshop, the Implementation Logic Model and Implementation Package were further refined.

#### 3.5.1 Coordination of the programme

Stakeholders discussed the specific tasks required to implement the programme, these included choosing dates, creating an advert, contacting participants and printing resources. It became clear that there was a need to distinguish between coordination and administration tasks. One delivery partner coordinator stated that they recognised the importance of having specific administrative support to successfully deliver the programme: ‘Admin is a separate role and is essential for the successful delivery of HPC’.

It was apparent that providing the organisations with the manuals and introduction to the programme through the initial workshops and premeeting was insufficient to manage concerns and expectations around the implementation of the programme, independent of the study team. One workshop participant stated that, ‘There was a lot of anxiety from the facilitators about the newness of the programme and the coordinators felt unsure about their roles and what was required’. Delivery partner staff also reported that they did not always feel able to make autonomous decisions, and noted the importance of ‘feeling empowered to make decisions around budgets, paperwork, date, etc’.

#### 3.5.2 Knowledge of the programme

Delivery partner staff noted that in‐depth knowledge was required to ensure successful implementation. For example, one manager commented that it was important to be able to ‘Identify appropriate facilitators with the necessary skills and competencies’, which required a level of knowledge about the programme to find the appropriate people.

#### 3.5.3 Governance

The importance of maintaining fidelity to the model and quality assurance was discussed by delivery partner staff, who expressed that it needed to be, ‘ensured that the knowledge of the programme stays “in house”’. Participants also noted the need for governance processes to be clear; for example, one member of the delivery partner organisation noted that organisationally there needed to be a consideration around the ‘quality assurance of delivery and training’.

#### 3.5.4 Strategic direction

The cost of delivering the programme on an ongoing basis and how it aligned to the strategic aims of their organisation was discussed by senior management, who explained the need to ‘prioritise the opportunity and cost [of running the programme] and how to situate it within its strategic aims’.

Consideration was given to the longer‐term sustainability of the programme, with a senior manager commenting, that ‘[they needed to continue] monitoring whether the programme is self‐sustaining’. They also noted that they needed to ensure the programme was sustainable from a resource and cost perspective: ‘[we need to] secure funding to support delivery and ensure it's over and above the minimum needed’.

### 3.6 Workshop 4: Sustainability strategies for a wider rollout

Workshop 4 presented an opportunity for a wider group of stakeholders to express views around the sustainability, hopes and barriers of delivering the programme now and in the future. It was attended by 14 participants, including three members of the research team, one commissioner, the Head of Service for Disabled Children, two members of an independent nonprofit organisation, two Parent Carer Forum chairs, the Business Development Manager from the University of Exeter, and the Director of Participation of Contact, and the Principal Officer of the CDC (see Table 2).

During the workshop participants discussed how impactful the programme could be for parent carers. One facilitator discussed the transformational effects it had on parents. The commissioner perceived the potential impact of the programme in potentially reaching and benefiting a larger number of parent carers. Discussions also took place around the potential economic advantages of delivering the programme online, including the potential for less overheads such as the hiring of a venue, time and payments for travelling.

However, challenges were noted specifically around how to maintain quality assurance, while increasing the number of courses delivered. A senior member of one organisation discussed their enthusiasm to deliver the programme but was concerned about not losing the quality when delivering on a much larger scale. A key point raised during the workshop was the recognition of the time commitment for parent carers to be trained and to deliver the programme on an ongoing basis. Workshop members shared opinions on the potential challenges of identifying parent carers with enough time and commitment available to deliver the programme.

**Figure 3:**
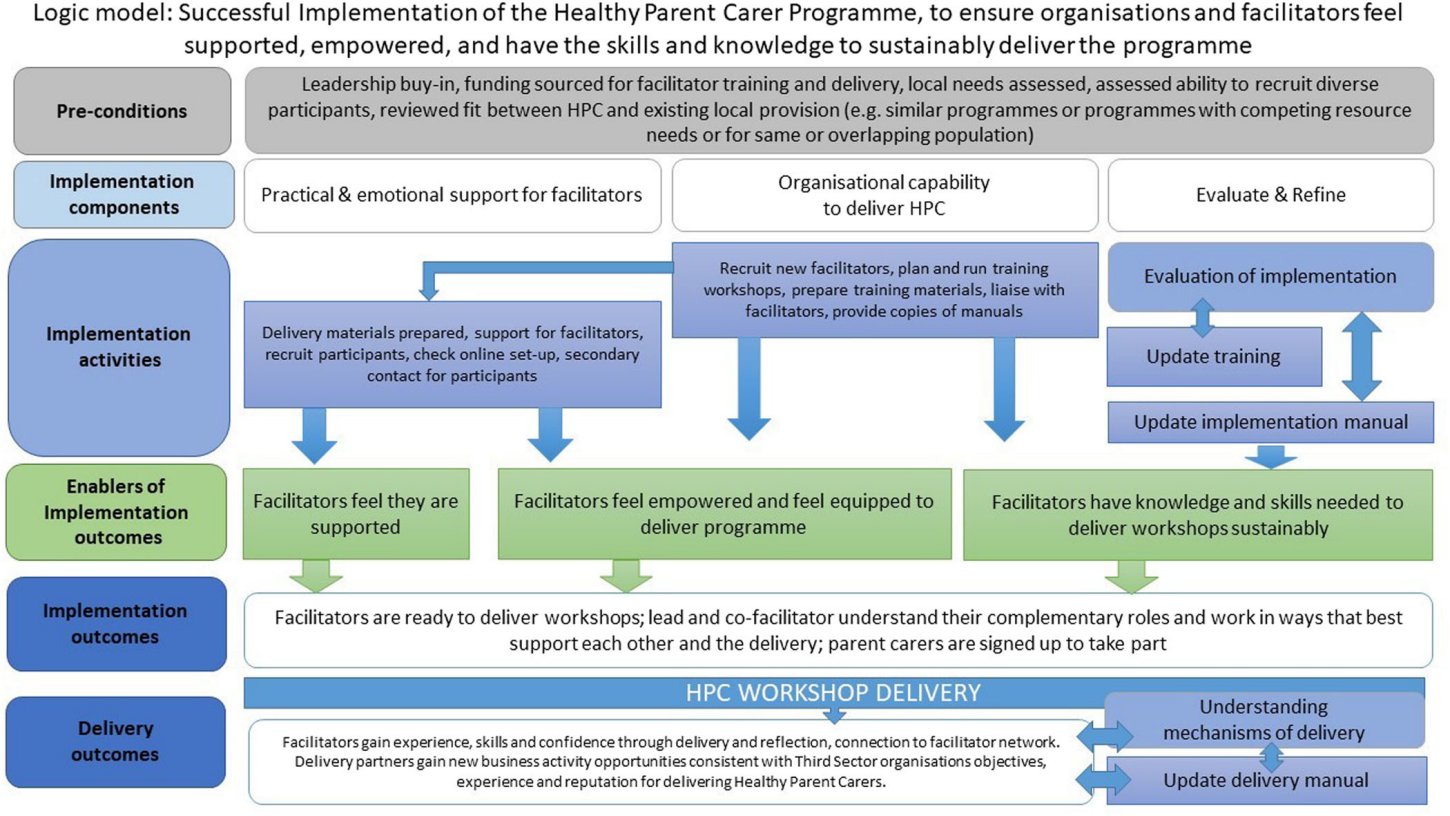
HPC Implementation Logic Model. HPC, Healthy Parent Carers.

Figure 3 is the final version of the HPC Implementation Logic Model that was developed iteratively over the course of the study. We found that the components associated with the successful implementation of the programme include ensuring that partner organisations felt empowered to deliver the programme by equipping them with the necessary skills, knowledge and expertise to enable delivery. The Implementation Logic Model indicates how organisational buy‐in, funding and assessment of existing provision and local needs are required to be in place before delivery partner organisations sign up to implement delivering HPC programme as well as access to the right skills mix of staff and level of expertise within their team. A shared understanding of the complementary roles, including strategic management, coordination and administration supported successful delivery. Practical considerations, such as the preparation of materials, access to appropriate recruitment mechanisms, such as *Eventbrite™* and a mailing list of parent carers, or social media links with parent carers proved to be effective recruitment strategies.

## 4 DISCUSSION

Using the initial phases of the REP framework, we engaged a wide range of stakeholders to explore the implementation of the HPC programme by a delivery partner organisation in the community rather than being managed by researchers. This is consistent with existing research, which shows following an evidence‐based formation such as REP is an easy and accessible framework which can help to support the identification of barriers and address implementation strategies.^32^ The two partner organisations involved in this study were able to recruit facilitators, participants and deliver the programme online to parent carers within different geographical regions and with participants from a wider range of ethnic backgrounds than in our earlier studies.^27^ Although the sample is small and thus the results are preliminary, feedback from staff, facilitators and participants was positive.

The study provides a further contextual understanding of the programme's implementation in different settings, taking a range of diverse perspectives, motivations and drivers into account. It provides key considerations for implementation at different levels, such as the importance of coordination and administration roles to support implementation activities effectively, and acknowledgement of the time commitments involved for delivery partner organisation personnel and facilitators, particularly when considering parent carers' responsibilities to ensure the future‐proofing of the programme and its implementation. It also highlights important governance considerations, including ensuring quality assurance to ensure fidelity when delivery is scaled up. The results also indicate the need for staff to be knowledgeable about the programme and its delivery when implementing it for the first time, potentially beyond a manualised format.

This study not only identified barriers and facilitators to implementation but also used that information to develop an implementation package that addresses these issues. Similar studies show that identifying and documenting effective strategies can help to improve uptake^33^ and increase the chances that the intervention is sustainable, scalable and adaptable to local service provision. It also highlights any specific local resources which may need to be priortised, and further provides a foundation from which the effectiveness of a scalable version of the programme can be tested.^34^, ^35^

The HPC programme was originally designed to be delivered in person. COVID‐19 presented both challenges but also a serendipitous opportunity to develop an online delivery version of the programme. Every aspect of the study was adapted to be delivered online, including facilitator training, workshops, meetings, recruitment, consent meetings and data collection. This provided invaluable learning around how to deliver the programme online and thus has changed our strategies around implementation moving forward.

An online format appears both an acceptable and practicable form of delivery. Online delivery provides a valuable alternative to in‐person delivery and potentially increases the programme's accessibility. For example, 7/16 participants, (44%) specifically reported that they had attended 10/12 sessions, compared to 57% of participants in our previous feasibility study, who attended 8/12 sessions. Furthermore, all participants in the current study reported attending at least one session, compared to the previous feasibility study, where 34% did not attend any sessions.^27^ Participants reported missing sessions for similar reasons, with the only addition of distance to travel being a reason for nonattendance in the in‐person feasibility study. These results could indicate that an online delivery format may be beneficial to parent carers to help increase their ability to attend sessions more easily. Nevertheless, we were mindful of potential safeguarding issues as the world moved online in the pandemic and took account of published recommendations on digital safeguarding principles.^36^ The online format also increases the sustainability and scalability of the programme by reducing the costs involved in face‐to‐face delivery, such as travel and venue hire, and may provide access to parent carers in remote areas or who cannot get to an in‐person group on a regular basis. However, further research will compare face‐to‐face and online delivery in terms of acceptability, engagement, and effectiveness. We are also considering how personal and contextual factors might influence engagement with the HPC programme and how we can ensure acceptability and equity, especially as online interventions may worsen inequality.^37^

In line with the findings from our previous study, participants reported that taking part in the programme helped to improve their health and wellbeing, and felt included and part of the group, suggesting that the specific strategies we adopted enabled the online groups to build positive connections.^38^ We believe that completing the programme modification work in collaboration with our Family Faculty public involvement group and giving attention to the group processes in the training and delivery manuals were key to maintaining these benefits.

### 4.1 Strengths and limitations

The strength of this research was that it followed systematically the REP framework, which provided an iterative, collaborative process with extensive stakeholder engagement to revise implementation and delivery strategies and processes in real‐world contexts.^29^

The current study has some limitations. The small number of delivery partners and participants involved is not necessarily representative of all potential delivery partners and eligible participants. In addition, research staff were more involved than initially intended. However, this is consistent with other studies during phases 1 and 2 of the REP framework.^39^ Further work to incrementally hand over responsibilities for training and delivery to delivery partner organisations is needed. In addition, future research across a larger number of and more diverse organisations, for example, local authorities and smaller delivery partner organisations, would allow us to continue to refine the implementation model for scalable rollout both nationally and internationally.

### 4.2 Implications and optimal implementation strategies

There is a growing field of parent‐carer‐focused interventions that either aim to teach parents about their child's condition, offer practical parental support, including advice and self‐care for their child's needs or self‐empowerment to interact with professionals.^40^, ^41^ However, none of these interventions specifically target the health and wellbeing of all parent carers. The HPC Programme was designed specifically in response to this need and gap in current provision.

Online recruitment seemed to work well and therefore similar strategies could be employed in the future to advertise and recruit to the programme. Other strategies that could be considered in future implementation included the use of Eventbrite™ and a template poster, which can be adapted by organisations. Specific consideration may be required in terms of the information provided in the advert, and the screening information collected via Eventbrite™, as this could help to ensure that people are adequately informed about the commitments involved and the aims and objectives of the programme. This may help to ensure higher retention rates. Advertising through online Parent Carer Forums provided a quick and effective means of recruitment; therefore, this method should be considered again when running future programmes. However, consideration should also be given around how to ensure parent carers who are not connected to these forums can be reached.

Despite organisations and facilitators having access to detailed manuals to support implementation, there was a lot of intrinsic knowledge required to run the programme. Facilitators and implementation staff preferred a dual approach, where information was provided both verbally, through in‐person meetings and through reading the manuals. Other comparable, REP‐based studies, similarly suggest that implementation with an independent, experienced facilitator, alongside standalone manuals could be a useful model to help community‐based organisations feel more confident to deliver, while they build up knowledge and further confidence to deliver the programme independently.^32^ Offering this approach potentially creates a more efficient implementation strategy and optimises early engagement, while allowing closer monitoring of the quality and fidelity of the programme. We will explore this as an option in the future evaluation of the programme. However, this does have an associated cost implication. The costs are likely to reduce over time, as materials and knowledge within organisations can be built upon and reused.

Evaluating the costs and benefits of running the programme is an important consideration in terms of its long‐term sustainability. Within the current study, two trainers, with equal responsibility, co‐delivered the training. However, since the programmes rollout, in the spring of 2022, this model changed to Lead and Assistant Trainers being employed with different remuneration rates. This is to optimise the likely affordability of the programme, as well as acknowledge the importance of modelling the different Lead and Assistant Facilitator roles. This model also creates training and employment opportunities for parent carers and may be more sustainable.^27^

## 5 CONCLUSION

Building on our earlier findings, which established satisfaction with the in‐person programme and programme and workshop participant reports of improved health and wellbeing, the current study demonstrated that it was feasible for trained staff from two different Delivery partner organisations to implement a programme developed by a research team.^27^, ^38^ This research suggests that delivering the programme online is a feasible and acceptable mode of delivery and potentially creates more accessibility and reach and may reduce costs. This study enabled the creation of a promising Implementation Package and logic model. Further evaluation with organisations from a wider range of contexts and sectors is now needed within an implementation, or hybrid implementation‐effectiveness trial.

## AUTHORS CONTRIBUTIONS

All authors contributed. Christopher Morris led the development programme and was the principal investigator. Alice Garrood managed the project, including overseeing day‐to‐day recruitment and data collection. Gretchen Bjornstad, Alice Garrood and Christopher Morris drafted the initial study design with input from Sarah Brand, Jenny Lloyd, Annabel McDonald, Mary Fredlund, Aleksandra Borek, Mark Tarrant, Annie Hawton, Vashti Berry and Stuart Logan. Aleksandra Borek, Annabel McDonald, Mary Fredlund and Christopher Morris designed the original programme. Annette Gillett, Annabel McDonald and Mary Fredlund revised the online version of the Facilitator Delivery Manual, with input from Aleksandra Borek, Christopher Morris, Gretchen Bjornstad and members of the Peninsula Childhood Disability Research Unit (PenCRU) Family Faculty. Aleksandra Borek coordinated the family's faulty stakeholder involvement. Sarah Brand designed the implementation elements and workshops along with Jenny Lloyd. Gretchen Bjornstad and Alice Garrood analysed the baseline and outcome data. Council for Disabled Children (CDC) and Contact recruited programme facilitators and arranged delivery sites. Annabel McDonald and Mary Fredlund planned, prepared and delivered facilitator training and support. CDC and Contact, with the support of Alice Garrood, recruited participants and facilitated data collection. All authors served on the Project Management Group, contributed to drafting this paper, and approved the final manuscript.

## CONFLICT OF INTEREST STATEMENT

The authors declare no conflict of interest.

## ETHICS STATEMENT

The University of Exeter Medical School Research Ethics Committee approved the study (UEMS REC 20/09/258). Delivery partner staff, facilitators and HPC programme participants all documented their consent to participate. Workshop 4 was a stakeholder consultation event so formal consent was not sought or documented.

## Supporting information

Supporting information.

Supporting information.

Supporting information.

## Data Availability

The data that support the findings of this study are available from the corresponding author upon reasonable request.

## References

[1] Barlow JH , Cullen‐Powell LA , Cheshire A . Psychological well‐being among mothers of children with cerebral palsy. Early Child Dev Care. 2006;176(3‐4):421‐428.

[2] Brehaut JC , Kohen DE , Raina P , et al. The health of primary caregivers of children with cerebral palsy: how does it compare with that of other Canadian caregivers? Pediatrics. 2004;114(2):e182‐e191. 15286255 10.1542/peds.114.2.e182

[3] Brehaut JC , Kohen DE , Garner RE , et al. Health among caregivers of children with health problems: findings from a Canadian population‐based study. Am J Public Health. 2009;99(7):1254‐1262. 19059861 10.2105/AJPH.2007.129817 PMC2696656

[4] Emerson E . Mothers of children and adolescents with intellectual disability: social and economic situation, mental health status, and the self‐assessed social and psychological impact of the child's difficulties. J Intellectual Disabil Res. 2003;47(4‐5):385‐399. 10.1046/j.1365-2788.2003.00498.x 12787168

[5] Lach LM , Kohen DE , Garner RE , et al. The health and psychosocial functioning of caregivers of children with neurodevelopmental disorders. Disabil Rehabil. 2009;31(9):741‐752. 19736648 10.1080/08916930802354948

[6] Murphy NA , Christian B , Caplin DA , Young PC . The health of caregivers for children with disabilities: caregiver perspectives. Child Care Health Dev. 2007;33(2):180‐187. 17291322 10.1111/j.1365-2214.2006.00644.x

[7] Oelofsen N , Richardson P . Sense of coherence and parenting stress in mothers and fathers of preschool children with developmental disability. J Intellect Dev Disabil. 2006;31(1):1‐12. 16766317 10.1080/13668250500349367

[8] Olsson MB , Hwang CP . Depression in mothers and fathers of children with intellectual disability. J Intellectual Disabil Res. 2001;45(6):535‐543. 10.1046/j.1365-2788.2001.00372.x 11737541

[9] Singer GHS . Meta‐analysis of comparative studies of depression in mothers of children with and without developmental disabilities. Am J Mental Retardation. 2006;111(3):155‐169. 10.1352/0895-8017(2006)111[155:MOCSOD]2.0.CO;2 16597183

[10] Tong C , Barest G . Approach to imaging the patient with neck pain. J Neuroimaging. 2003;13(1):5‐16. 12593126

[11] Lee MH , Park C , Matthews AK , Hsieh K . Differences in physical health, and health behaviors between family caregivers of children with and without disabilities. Disability Health J. 2017;10:565‐570. 10.1016/j.dhjo.2017.03.007 28347641

[12] Arim RG , Miller AR , Kohen DE , Guèvremont A , Lach LM , Brehaut JC . Changes in the health of mothers of children with neurodevelopmental disabilities: an administrative data study. Res Dev Disabil. 2019;86:76‐86. 30684833 10.1016/j.ridd.2018.12.007

[13] O'Dwyer ST , Janssens A , Sansom A , et al. Suicidality in family caregivers of people with long‐term illnesses and disabilities: a scoping review. Compr Psychiatry. 2021;110:152261. 34332205 10.1016/j.comppsych.2021.152261

[14] Berghs M , Atkin K , Graham H , Hatton C , Thomas C . Implications for public health research of models and theories of disability: a scoping study and evidence synthesis. Public Health Res. 2016;4(8):1‐166. 27512753

[15] Tong HC , Haig AJ , Nelson VS , Yamakawa KS , Kandala G , Shin KY . Low back pain in adult female caregivers of children with physical disabilities. Arch Pediatr Adolesc Med. 2003;157(11):1128‐1133. 14609905 10.1001/archpedi.157.11.1128

[16] Disabled Children's Partnership . Left in lockdown: parent carers' experiences of lockdown. p. 23. 2021. https://disabledchildrenspartnership.org.uk/wp-content/uploads/2020/06/LeftInLockdown-Parent-carers%E2%80%99-experiences-of-lockdown-June-2020.pdf

[17] Merrick H , Driver H , Main C , et al. Impacts of health care service changes implemented due to COVID‐19 on children and young people with long‐term disability: a mapping review. Dev Med Child Neurol. 2022;65:885‐899. 10.1111/dmcn.15503 36649197

[18] Alcaraz KI , Yanez BR . Interventions to promote health equity: implications for implementation science in behavioral medicine. Transl Behav Med. 2022;12(9):885‐888. 10.1093/tbm/ibac062 36205475 PMC9540972

[19] Borek AJ , McDonald B , Fredlund M , Bjornstad G , Logan S , Morris C . Healthy Parent Carers programme: development and feasibility of a novel group‐based health‐promotion intervention. BMC Public Health. 2018;18(1):270. 29458355 10.1186/s12889-018-5168-4 PMC5819077

[20] Hammond P . Staying Alive: How to Get the Best Out of the NHS—Advice From a Doctor. Quercus; 2015:320.

[21] Michie S , Richardson M , Johnston M , et al. The behavior change technique taxonomy (v1) of 93 hierarchically clustered techniques: building an international consensus for the reporting of behavior change interventions. Ann Behav Med. 2013;46(1):81‐95. 23512568 10.1007/s12160-013-9486-6

[22] Borek A , Abraham C , Greaves C , et al. Identifying change processes in group‐based health behaviour‐change interventions: development of the Mechanisms of Action in Group‐Based Interventions (MAGI) framework. Health Psychol Rev. 2019;13:227‐247. 31190619 10.1080/17437199.2019.1625282

[23] Cane J , Richardson M , Johnston M , Ladha R , Michie S . From lists of behaviour change techniques (BCTs) to structured hierarchies: comparison of two methods of developing a hierarchy of BCTs. Br J Health Psychol. 2015;20(1):130‐150. 24815766 10.1111/bjhp.12102

[24] Marchand E , Stice E , Rohde P , Becker CB . Moving from efficacy to effectiveness trials in prevention research. Behav Res Ther. 2011;49(1):32‐41. 21092935 10.1016/j.brat.2010.10.008 PMC3883560

[25] Glasgow RE , Lichtenstein E , Marcus AC . Why don't we see more translation of health promotion research to practice? Rethinking the efficacy‐to‐effectiveness transition. Am J Public Health. 2003;93(8):1261‐1267. 10.2105/AJPH.93.8.1261 12893608 PMC1447950

[26] Skivington K , Matthews L , Simpson SA , et al. A new framework for developing and evaluating complex interventions: update of Medical Research Council guidance. BMJ. 2021;374:n2061. 34593508 10.1136/bmj.n2061 PMC8482308

[27] Bjornstad G , Cuffe‐Fuller B , Ukoumunne OC , et al. Healthy Parent Carers: feasibility randomised controlled trial of a peer‐led group‐based health promotion intervention for parent carers of disabled children. Pilot Feasibility Stud. 2021;7:144. 34301334 10.1186/s40814-021-00881-5 PMC8298691

[28] Nápoles AM , Santoyo‐Olsson J , Stewart AL . Methods for translating evidence‐based behavioral interventions for health‐disparity communities. Prev Chronic Dis. 2013;10:130133. 10.5888/pcd10.130133 PMC3839588 24262025

[29] Kilbourne AM , Neumann MS , Pincus HA , Bauer MS , Stall R . Implementing evidence‐based interventions in health care: application of the replicating effective programs framework. Implementation Science. 2007;2(1):42. 18067681 10.1186/1748-5908-2-42 PMC2248206

[30] Williams U , Rosenbaum P , Gorter JW , McCauley D , Gulko R . Psychometric properties and parental reported utility of the 19‐item ‘About My Child’ (AMC‐19) measure. BMC Pediatr. 2018;18(1):174. 29801450 10.1186/s12887-018-1147-2 PMC5968543

[31] Department for Communities and Local Government . English indices of deprivation. 2015. Accessed May 12, 2017. https://www.gov.uk/government/statistics/english-indices-of-deprivation-2015

[32] Smith SN , Almirall D , Prenovost K , et al. Change in patient outcomes after augmenting a low‐level implementation strategy in community practices that are slow to adopt a collaborative chronic care model: a cluster randomized implementation trial. Med Care. 2019;57(7):503‐511. 31135692 10.1097/MLR.0000000000001138 PMC6684247

[33] Kirchner JE , Smith JL , Powell BJ , Waltz TJ , Proctor EK . Getting a clinical innovation into practice: an introduction to implementation strategies. Psychiatry Res. 2020;283:112467. 31488332 10.1016/j.psychres.2019.06.042 PMC7239693

[34] Flay BR . Efficacy and effectiveness trials (and other phases of research) in the development of health promotion programs. Prev Med. 1986;15(5):451‐474. 3534875 10.1016/0091-7435(86)90024-1

[35] Beidas RS , Saldana L , Shelton RC . Testing psychosocial interventions in the contexts they are meant to be delivered. J Consult Clin Psychol. 2023;91:189‐191. 36780266 10.1037/ccp0000797 PMC10175148

[36] Abuse AVa . Digital safeguarding resource pack. p. 50. 2020. https://avaproject.org.uk/wp-content/uploads/2020/05/AVA-Digital-Resource-Pack-Updated-2021.docx.pdf

[37] Veinot TC , Mitchell H , Ancker JS . Good intentions are not enough: how informatics interventions can worsen inequality. J Am Med Inform Assoc. 2018;25(8):1080‐1088. 29788380 10.1093/jamia/ocy052 PMC7646885

[38] Lloyd J , Bjornstad G , Borek A , et al. Healthy Parent Carers programme: mixed methods process evaluation and refinement of a health promotion intervention. BMJ Open. 2021;11(8):e045570. 10.1136/bmjopen-2020-045570 PMC8388296 34433591

[39] Boucher NA , Zullig LL , Shepherd‐Banigan M , et al. Replicating an effective VA program to train and support family caregivers: a hybrid type III effectiveness‐implementation design. BMC Health Serv Res. 2021;21:430. 33952263 10.1186/s12913-021-06448-7 PMC8099701

[40] Miller L , Nickson G , Pozniak K , et al. ENabling VISions and Growing Expectations (ENVISAGE): parent reviewers' perspectives of a co‐ designed program to support parents raising a child with an early‐onset neurodevelopmental disability. Res Dev Disabil. 2022;121:104150. 34942443 10.1016/j.ridd.2021.104150

[41] Coulman E , Gore N , Moody G , et al. Early positive approaches to support (E‐PAtS) for families of young children with intellectual disability: a feasibility randomised controlled trial. Front Psychiatry. 2021;12:729129. 34992552 10.3389/fpsyt.2021.729129 PMC8725992

