# Supplementary material for: Healthy Parent Carers: Acceptability and practicability of online delivery and learning through implementation by delivery partner organisations
Source: Health Expect. 2023 Jul 4;26(5):2050–63. doi: 10.1111/hex.13812 (PMC10485339; doi:10.1111/hex.13812)
Supplement: Supplementary file 1 — Supporting information. [file HEX-26--s001.docx]

**Supplementary file 1: Adaptation for Online Delivery**

We reviewed the Facilitator Delivery Manual for in-person delivery with the aim of identifying activities that may be impossible or difficult to deliver online. We used a traffic light system to assess the extent to which each activity appeared challenging to be delivered online. For those activities that were considered challenging, we identified possible adaptations or alternative activities that would be more feasible for delivering in online groups, while aiming to achieve the same objectives and maintaining fidelity to the original model. A working group with parent carers from our Family Faculty met for six online meetings to test out the tasks online, and to discuss the adaptations. Activities that were deemed feasible for online delivery were adopted. Key changes made to the Delivery Manual are summarised in Table 2.

An Online Facilitator Training Manual was also created, which included the specifically modified online activities and tips for online delivery. The length and number of sessions of the online training format was also reviewed, to take into account potential concentration fatigue using an online format. Learning from the online training subsequently informed anew Facilitator Training Manual.

**Table:** Key revisions in the Online Delivery Manual

| Delivery Manual item | Specific manual change/adaptation |
| --- | --- |
| Instructions | - ‘Top Tips’ for online delivery were added to the introduction, with detailed instructions around how to use specific online platforms required for the programme and information on how to manage online group dynamics and facilitation­­­­ - Addition of a box stating required Zoom™ functions for each activity - Addition of a set of online group rules and information around how to manage risk online |
| Format | - Inclusion of specific online platforms – Zoom™ and Jamboard™ - For ease of use, the activities were written using a two-page open-book format so facilitators do not need to turn pages during an activity - Optimum number of participants was specified |
| Activities | - Online-friendly icebreakers/introductions - Use of breakout rooms - Change of specific activities to fit online format (e.g. for the ‘Juggling Responsibilities’ activities, participants required a toilet roll and cup rather than balloons) |
