# Supplementary figures and images for: Healthy Parent Carers: Acceptability and practicability of online delivery and learning through implementation by delivery partner organisations
Source: Health Expect. 2023 Jul 4;26(5):2050–63. doi: 10.1111/hex.13812 (PMC10485339; doi:10.1111/hex.13812)

**Supplementary file 2:** MIRO™ board outputs from Workshop 3: Implementation Staff Experience


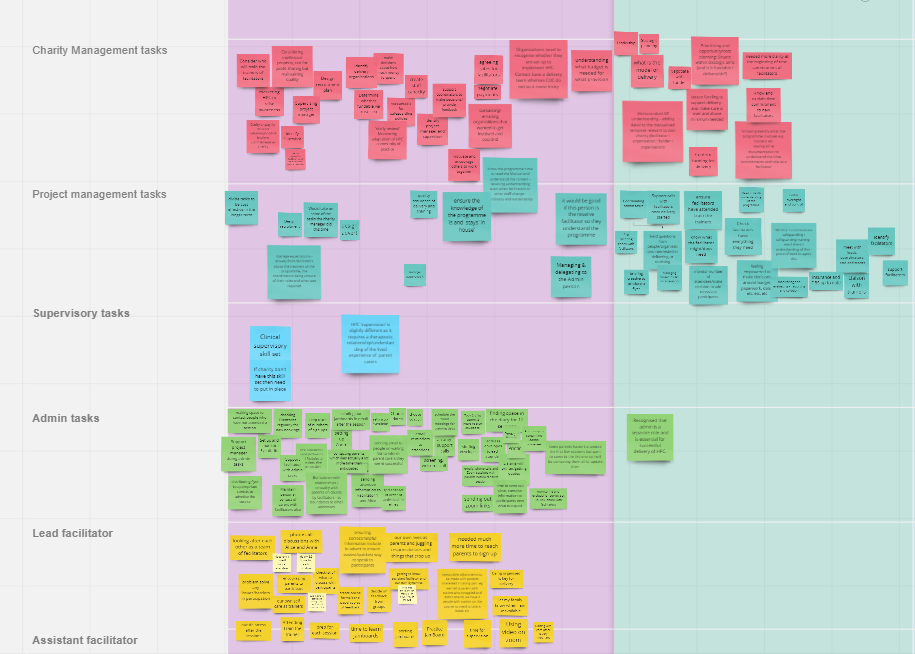

Supplement: Supplementary file 2 — Supporting information. [file HEX-26--s002.docx]
