# Supplementary material for: Healthy Parent Carers: Acceptability and practicability of online delivery and learning through implementation by delivery partner organisations
Source: Health Expect. 2023 Jul 4;26(5):2050–63. doi: 10.1111/hex.13812 (PMC10485339; doi:10.1111/hex.13812)
Supplement: Supplementary file 3 — Supporting information. [file HEX-26--s003.docx]

**Supplementary Materials**

**Table S1:** Baseline demographics of study participants

| **Characteristic** | **Participants N = 16** |
| --- | --- |
| Female, n (%) | 15 (94) |
| Age, mean (SD) | 44.1 (3.9) |
| Number of children |  |
| One, n (%) | 3 (19) |
| Two, n (%) | 10 (63) |
| Three, + n (%) | 3 (19) |
| Female index child, n (%) | 6 (38) |
| Age of index child, mean (SD) | 10.9 (3.7) |
| Relationship status |  |
| Married, or Civil Partnership (%) | 10 (63) |
| Single, n (%) | 3 (19) |
| Divorced, separated or widowed n (%) | 3 (19) |
| Ethnicity |  |
| White, n (%) | 6 (38) |
| Asian/Asian British, n (%) | 6 (38) |
| Black/African/Caribbean/Black British, n (%) | 4 (25) |
| Employment status |  |
| Working full, or part-time, n (%) | 4 (25) |
| Unemployed, n (%) | 11 (69) |
| Not reported, n (%) | 1 (6) |
| Educational qualification |  |
| 1 to 5+ GCSEs/equivalent, n (%) | 3 (19) |
| 2+ A levels, or Degree n (%) | 10 (63) |
| No qualifications, other n (%) | 3 (19) |
| Total household income per week |  |
| Less than £100 to £299, n (%) | 4 (25) |
| £300 to £499, n (%) | 3 (19) |
| £500 to £999, n (%) | 5 (31) |
| £1000 or more | 2 (13) |
| Not reported, n (%) | 2 (13) |
| Housing tenure |  |
| Owned outright, shared ownership, or mortgage/loan n (%) | 7 (44) |
| Privately rented, n (%) | 3 (19) |
| Council rented, other social rented n (%) | 5 (31) |
| Not reported, n (%) | 1 (6) |
| IMD* 2019, quintile-based group |  |
| Most deprived, n (%) | 3 (19) |
| 2^nd^ group, n (%) | 7 (44) |
| 3^rd^ group, n (%) | 2 (13) |
| 4^th^ group, n (%) | 2 (13) |
| Least deprived, n (%) | 2 (13) |
| AMC** impact score (index child), mean (SD) | 53.3 (10.2) |

* IMD Index of Multiple Deprivation ^28^

** AMC About My Child ^27^

**Supplementary document: End of Programme Feedback Questionnaire**

**HEALTHY PARENT CARERS**

**Feedback Questionnaire**

| We would like to know what you think about the Healthy Parent Carers programme and would be grateful for your feedback. The feedback is confidential, and it will help us refine and improve the programme. |
| --- |

1. **How did you hear about the Healthy Parent Carer Programme?**

| Parent Carer Forum (please state which) | Via Contact, e.g. Website or other (please specify) | Via CDC, e.g. Website or Other (please specify) | Other (please specify where) |
| --- | --- | --- | --- |
|  |  |  |  |

1. **Was the initial pre-meeting you had with the facilitator before the group started helpful in making you feel comfortable to attend?**

No / Yes

1. **Did you attend any of the sessions?**

No / Yes

***If yes, go to question 5***

***If no, to question 4***

1. **If not, why were you unable to attend?**

| The sessions were at a time that I couldn’t make | Something else came up to stop me being able to attend | I changed my mind | Another reason - please tell us why |
| --- | --- | --- | --- |
|  |  |  |  |

1. **Were you happy to access an online group?**

| Yes, I wouldn’t have been able to attend an in- person group | Yes | I would have preferred to attend an in person group | No, or other (please specify) |
| --- | --- | --- | --- |
|  |  |  |  |

**6. Did you have any problems with your internet connection, which prevented you from accessing the group at all?**

Yes / No

If yes, what?

|  |
| --- |

**7. Did you look at the resources sent to you before the group started?**

Yes / No

**8. Did this change how you felt about attending?**

Yes / No

If yes, what?

|  |
| --- |

**9. Did you have the resources you needed to be able to participate? (e.g pens, print outs, sticky notes etc) Where you told what you needed in advance of each session?**

Yes / No

If no, why?

|  |
| --- |

**10. Were able to, and confident accessing the online platforms used in the groups (Zoom, JamBoard etc)?**

Yes / No

If no, why?

|  |
| --- |

**11. How would you rate the length of the individual sessions?**

| Too short | About right | Too long |
| --- | --- | --- |
|  |  |  |

1. **How would you rate the times and dates of the sessions?**

| Fine/OK | Would prefer a different time of day | Would prefer a different day of the week |
| --- | --- | --- |
|  |  |  |

1. **Did you miss any of the group sessions?** Yes / No

If yes, why?

|  |
| --- |

Any other comments:

**14. Do you have any suggestions about how the programme or materials could be improved?**

|  |
| --- |

1. **Overall, how satisfied are you with taking part in the Healthy Parent Carers programme?**

Not satisfied at all Very satisfied

1 2 3 4 5

1. **How useful has the programme been in helping you improve your health and wellbeing?**

Not at all Very much

1 2 3 4 5

Was there anything in particular that helped you?

|  |
| --- |

1. **Have you made any changes as a result of taking part in the programme?** No / Yes

If yes, what?

|  |
| --- |

If no, why?

|  |
| --- |

**18. Overall, how much did you feel included and part of the group?**

Not at all Very much

1 2 3 4 5

**19. How satisfied were you with the way the sessions were facilitated and delivered?**

Not satisfied at all Very satisfied

1 2 3 4 5

**20. Would you recommend this programme to other parent carers?**

Not at all Very much

1 2 3 4 5

**21. Do you have any other comments, reflections or suggestions?**

|  |
| --- |

Many thanks for completing.
